# Adherence to Glyburide and Metformin and Associated Factors in Type 2 Diabetes in Isfahan, Iran

**Published:** 2011

**Authors:** Shadi Farsaei, Ali Mohammad Sabzghabaee, Amir Hooshang Zargarzadeh, Massoud Amini

**Affiliations:** a *Department of Clinical Pharmacy and Pharmacy Practice, School of Pharmacy and Pharmaceutical Sciences, Isfahan University of Medical Sciences, Isfahan, Iran.*; b *Isfahan Clinical Toxicology Research Center, Isfahan University of Medical Sciences, Isfahan, Iran.*; c *Isfahan Endocrinology and Metabolism Research Centre, Isfahan University of Medical Sciences Center, Isfahan, Iran. *

**Keywords:** Adherence, Pill count, Self report, Type 2 diabetes, Hemoglobin A_1C_

## Abstract

The purpose of this study was to determine the adherence to oral hypoglycemic medications and associated factors in type 2 Diabetes Mellitus patients who were referred to the Isfahan Endocrinology and Metabolism Research Centre (IEMRC). Convenience sampling was used to enroll 248 patients with type 2 diabetes in a prospective study at IEMRC from January 2007 to January 2008. Patients had to be on a stable dose of oral hypoglycemic medications (glyburide and metformin) or for 3 months prior to the study and willing to participate in consultation sessions with a pharmacist. Pill count and self report methods were used to measure the adherence. Mean (SD) of patients studied was 56.6 (8.9) years and 62% were females. The mean (SD) duration of diabetes in the study patients was 10.8 (6.1) years and 81.9% of them were literate with basic education. Non-adherence rates to metformin and glyburide were recorded in 39.7% and 35.3% of the study population respectively. Lower HbA_1C_ levels and higher education were associated with higher adherence rates. Forgetfulness, confusion, fasting, adverse effects, complexity of medication regimen and disruption of routines were most commonly reported causes of non adherence. Prevalence of adherence to these two medications did not differ significantly between pill count (62.3%) and self report (62.8%), (p >0.05). Adherence rates did not vary by pill count and self report significantly. It was concluded that good adherence to medications was associated with a lower HbA_1C_ profile; so it seems that pill count is a useful method in the clinical practice to identify non-adherent patients. Further studies are needed to find out efficient interventions to improve the patient’s adherence.

## Introduction

Diabetes mellitus is a chronic disease which requires life-long therapy (1, 2). Glycemic control has a main role in diabetes management (3). Achievement of glycemic control depends mainly upon patient adherence to the treatment plan. Adherence is the default medical term used in literature to depict patient’s behavior (in terms of taking medication, following diets or executing lifestyle changes) (4). Adherence to taking medication, following diet and executing life style changes for managing diabetes is essential to achieve the goal of metabolic control and decrease total annual health care costs (5, 6) Adherence to a medication regimen has been defined as the extent that each patient takes medications as prescribed by their health care providers (7).

There are different methods to measure adherence to oral hypoglycemic agents (OHAs) like pill count, Medication Event Monitoring System (MEMS), refill data and self report (8). 

Non-adherence to OHAs may lead to suboptimal therapeutic goals and also associated with increased risk of hospitalization (9-11). Although medication adherence is very important for reaching glycemic control and reducing complications, previous studies have shown that people with diabetes do not use their medications as prescribed (4, 12). A systematic review of literature showed that adherence to OHAs which measured by retrospective analysis ranged from 36-93%, although prospective electronic monitoring analysis of outcomes documented 67-85% of patients took OHAs as prescribed (4). 

Previous studies have reported the role of different factors as predictors of medication adherence such as patient characteristics, complexity of the therapeutic regimen and characteristics of health care systems (13-16).

Research on adherence has focused mainly on the barriers patients face in adhering to medication regimens. Barriers to adherence are categorized in to different aspects: 1- social and economic, 2- health care team, 3- condition, 4- therapy, 5- patient related factors (7, 17). 

Although several studies were carried out in developed countries, there is a gap in information about adherence in developing countries such as Iran (4, 18, 19).

*Aim of this study*

In the present study we measured adherence to OHAs by pill count and self report. Furthermore, barriers and factors associated with adherence were assessed for type 2 diabetes patients in Isfahan Endocrinology and Metabolism Research Centre (IEMRC).

## Experimental

*Materials and methods*

We have conducted a prospective clinical study of patients with type 2 diabetes followed in IEMRC, the largest diabetes clinic in Isfahan. Because in Iran medication refill data are not registered in community pharmacies and electronic devices such as medication electronic monitoring system (MEMS) are not available for measurement of adherence to medication, self report and pill count were used to evaluate patient›s adherence.

*Setting*

IEMRC is the outpatient diabetic clinic where medical care is provided throughout the week. Patients are educated and followed up at least every three months for their diabetes control; also they participate in research programs in this center.

*Inclusion-exclusion criteria*

Patients who were not on a stable dose of either medication for the past three months prior to their initial visit, those who did not have a documented HbA_1C_ value within the 90 day period after their initial visit with the pharmacist in IEMRC and those who did not keep their scheduled visit 3 months after their initial visit were excluded. 

*Data collection and measures*

First a pilot study was conducted in January 2007 to determine problems which may occur during the study, modify the survey instrument and establish a trust relationship between patients and the pharmacist. Then a convenience sample of 248 type 2 diabetic patients between 35-75 years old were enrolled in the study from June to September 2007 after obtaining patient’s informed consent. Patients had to have at least one prescription for one of the oral hypoglycemic agents, glyburide or metformin, filled in IEMRC. 

The pharmacist (primary author) interviewed patients who were waiting to visit their physician and filled a survey including patient demographics (age, sex, BMI, education), duration of diabetes, laboratory test results (HbA_1C_, FBS) and medication information. 

After considering ethical issues, patients who met the inclusion criteria were introduced to the attending physician. Necessary information regarding dose and number of prescribed OHAs (metformin or glyburide) were filled out by the pharmacist. Patients were asked to return their unused medications at the time of next visit. When patients returned for the next scheduled visit, adherence would be calculated by pill count and self report. Patients were asked how they had been taking their medication during the last three months and reasons for not taking their medications were questioned. HbA_1C_ was measured at the end of the three months to evaluate the relationship between diabetes control and adherence. 

*Assessment of adherence*

*Pill count*

Pill count was conducted when the patients returned for the second visit by the pharmacist. Adherence ratio assessed by pill count was defined as: number of medicines taken during the three month period divided by the number of medicines that should have been taken during the same period (expressed in %). 

*Adherence*

Patients who took between 90 and 105% of their medication (90% < adherence ratio < 105%) considered as adherent and patients that took < 90% or > 105% of their medications were classified as non-adherent (19-22).

*Self report*

patients were asked to describe their medication behavior during the last three months. Patients who reported taking less than 90% or more than 105% of their prescribed diabetes medicines were considered not to be adhering to their treatment. Information about self-reported barriers to adherence was asked using open-ended questions but was later coded. 

*Statistical analysis*

Continuous quantitative data with a normal distribution were expressed as mean ± standard deviation (SD). In statistical analysis, descriptive statistics were used to obtain demographic and clinical characteristics of study participants and the prevalence of non-adherence to oral hypoglycemic agents. An unpaired student’s t-test was performed to compare continuous quantitative data between adherent patients versus non-adherent patients and patients with controlled HbA_1C_ (≤ 7%) versus patients with uncontrolled HbA_1C_ (> 7%). Chi Square analysis was used to describe the relation between reaching HbA_1C_ goal and adherence in addition to identify differences in gender in each group. The non-parametric rank test according to Mann-Whitney was used to compare the distribution of education ranks between the groups. Two-tailed p-value < 0.05 was considered statistically significant and data processing were carried out by SPSS statistical software version 13.0.

*Ethical issues*

each patient after informed consent enrolled in the study. 

## Results

Two hundred forty eight patients (371 medications) met the inclusion-exclusion criteria for final analysis. The patients responded to interview about demographic-clinical characteristics and adherence questionnaire. 

Eligible patients were aged 56.6 (± 8.9) )mean ± )standard deviation() years and over half of them were female (62.0%). They were predominantly literate with a basic education (81.9%). Patients had a mean duration of diabetes of 10.8 (± 6.1) years and a mean body mass index (BMI) of 28.8 (± 4.1) kg/m^2^. The average fasting blood glucose and HbA_1C _were 160.4 mg/dL (± 51.3) and 7.4% (± 1.3) respectively. We identified 36.0% of patients reached an HbA_1C _goal of ≤7 % and half of them had been prescribed both metformin and glyburide.

Prevalence of adherence of 371 patients taking oral hypoglycemic agents was considered 62.3% based on pill count and according to self report 62.8% of patients were non-adherent to oral hypoglycemic agents.

According to pill count 60.3% of 204 patients taking metformin and 64.7% of 167 patients taking glyburide were adherent to their medication. Self report of taking medication showed that 57.2% and 69.5% of patients were adherent to metformin and glyburide respectively. 

**Figure 1:**
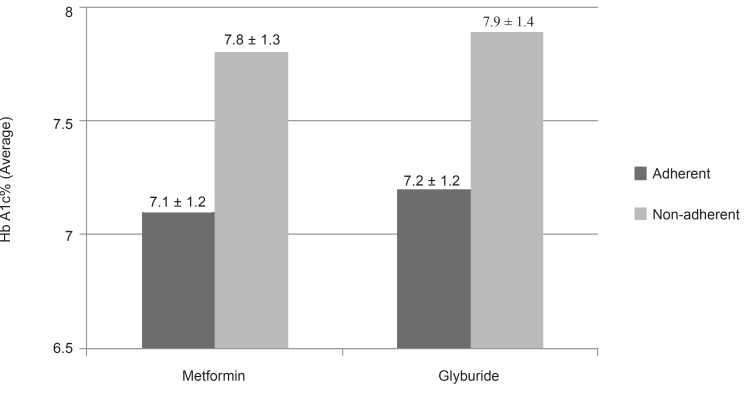
Mean HbA_1C_ (with confidence 95% interval bars) of patients being and not being adherent to oral hypoglycemic agent

The effect of patient clinical and demographic characteristics on adherence were also evaluated and we found a significant relation between lower HbA_1C_ and being adherent. The mean HbA_1C _of adherent patients was statistically lower than those who not being adherent (p < 0.05) (Figure 1). The only demographic factor significantly associated with adherence was education (p = 0.007), adherent patients had higher education level than non-adherent patients. Other demographic factor such as age, gender and duration of diabetes treatment did not affect adherence to medication significantly.

The mean of adherence to ratio for OHAs among patients reached an HbA_1C_ goal of ≤ 7% was statistically more than those who did not reach the goal (p < 0.05). chi-square test showed a significant association between reaching HbA_1C_ goal ≤ 7% and being adherent (p < 0.05) (Table1). 

**Table 1:** Relationship between the adherence to oral hypoglycemic agents (OHAs) and HbA_1C_ in type 2 diabetic patients recruited in IEMRC from June to September 2007

**Characteristic **		**Adherence to OHAs**		p-value *
		Adherent	Non-adherent	
HbA_1C_	>7%	121(55.3%)	98 (73.1%)	0.001
	≤7%	98 (44.7%)	36 (26.9%)	

* chi-square test

The most common reported barriers to adherence with oral anti-hyperglycemic medications were: forgetting to take doses (38%), confusion (15.1%), fasting in Ramadan (11%), adverse effects (8%), complexity of the therapeutic regimen (4.5%) and disruption of routines (3.5%).

## Discussion

This study evaluated the adherence to OHAs and also relation between adherence and HbA_1C_ in type-2 diabetic patients enrolled in IEMRC. The present study revealed that 62.3% of patients were adherent to OHAs. Previously conducted studies showed different ranges of adherence measured with pill count. These differences may be related to the study setting and population. 

Winkler *et al. *reported similar results to our study which used a similar study population who were voluntary patients observed over two months in a diabetic center. Also we used the same method and definition for adherence measurement. In this study Adherence to oral hypoglycemic medications measured with pill count was 57.9%. (19). 

Other conducted studies also reported high adherence (71%) when measured adherence by the pill count method (13, 20). Adherence of our study population was high when compared to adherence of diabetic patients in Mexico which was 27.0%. In this study Prado-Aguilar *et al*. used home visiting for pill count which may calculate adherence more precise than our study (20). Furthermore our patients were educated about diabetes control which could increase their adherence.

In our study, adherence to glyburide was more than metformin; because patients thought that, glyburide is more effective than metformin for achieving glycemic control. This misconception may be related to the adverse effect of glyburide (hypoglycemic).

HbA_1C_ was significantly lower in patients who reached adherence goal than patients who did not reach the target adherence goal. 

There are previously published reports that demonstrated adherence are related to better glycemic control but independent from method of measurement, study population and setting (13, 21, 23, 24, 25). For example Pladevall *et al. *showed non-adherence was clinically and statistically associated with worse outcomes. They concluded that better glycemic control was related to greater medication adherence (26). We found similar results to other conducted studies which the higher medication adherence is related to lower HbA_1c_ values. 

Also a significant association between education level and adherence is attained. Patients with lower level of education are at greater risk for non-adherence than patients with higher level of education; so an educational intervention may be useful for resolving non-adherence problem among patients with low level of education.

Self report was also used for measurement of adherence. The relatively high proportion of participants (62.8%) were adherent to medication according to self report and reported no barriers to adherence. In our study, adherence based on pill count and self report were related quite well. This may show that reported barriers to adherence affect adherence measured by pill count.

Forgetting to take doses, confusion and fasting in Ramadan were the most common reported barriers to adherence. By considering the barriers to adherence, an interventional program can be designed to improve the patient’s ability to follow a medication regimen. Patient education and interaction between patient and health care team will have the greatest effect on improving medication adherenc (2).

It would be necessary to establish a pharmacy record system in community pharmacies for measuring adherence to medication more precise. Future studies might be performed to focus on the more accurate measure of adherence to medication and also consider factors related to adherence in the large population of diabetic patients.

*Limitations*

First, it is supposed that the level of adherence in this population is an overestimation of true value of adherence in the society. 

Patients in this population should be followed-up every three months for measuring of adherence, so they were selected from IEMRC outpatient clinic. IEMRC cover more than 90% of diabetic population of Isfahan and follow patients at least every three months. 

We estimated that, the population study might be expected to be more adherent compared to other diabetic patients in the society, because: these patients received educational program and voluntarily participated and completed this study, presence of pharmacist during pill count may motivate them to take their medication as prescribed

Second, it should be mentioned that because we do not have any documented pharmacy record about taking the medication, adherence was determined by pill count. Some published studies suggest the overestimation of adherence by pill count, because participants learned to return the appropriate number of pills to appear adherent (19, 22).

## Conclusion

To our best of knowledge, no such data of Iranian diabetic patients have been published yet. Significant relationship emerged between adherence to medication and lower related clinical outcomes showed that pill count can be useful to identify high risk patients for non-adherence. Adherence to medication predicted the clinical outcome of diabetes support development and evaluation of intervention for improving medication adherence. 
